# Supplementary material for: Role and mechanism of NCAPD3 in promoting malignant behaviors in gastric cancer
Source: Front Pharmacol. 2024 Apr 22;15:1341039. doi: 10.3389/fphar.2024.1341039 (PMC11070777; doi:10.3389/fphar.2024.1341039)
Supplement: Supplementary file 11 [file DataSheet2.ZIP › GSEA/immunologic/my_analysis.Gsea.1599462003817/index.html]

Index for xtools.gsea.Gsea my\_analysis.Gsea.1599462003817

### GSEA Report for Dataset filtered\_dataset

#### Enrichment in phenotype: **WT (3 samples)**

- 1302 / 3681 gene sets are upregulated in phenotype **WT**- 0 gene sets are significant at FDR < 25%- 20 gene sets are significantly enriched at nominal pvalue < 1%- 60 gene sets are significantly enriched at nominal pvalue < 5%- Snapshot of enrichment results- Detailed enrichment results in html format- Detailed enrichment results in TSV format (tab delimited text)- Guide to interpret results

#### Enrichment in phenotype: **NCAPD3\_MUT (3 samples)**

- 2379 / 3681 gene sets are upregulated in phenotype **NCAPD3\_MUT**- 590 gene sets are significantly enriched at FDR < 25%- 244 gene sets are significantly enriched at nominal pvalue < 1%- 526 gene sets are significantly enriched at nominal pvalue < 5%- Snapshot of enrichment results- Detailed enrichment results in html format- Detailed enrichment results in TSV format (tab delimited text)- Guide to interpret results

#### Dataset details

- The dataset has 1411 features (genes)- No probe set => gene symbol collapsing was requested, so all 1411 features were used

#### Gene set details

- Gene set size filters (min=15, max=500) resulted in filtering out 1191 / 4872 gene sets- The remaining 3681 gene sets were used in the analysis- List of gene sets used and their sizes (restricted to features in the specified dataset)

#### Gene markers for the **WT** *versus* **NCAPD3\_MUT** comparison

- The dataset has 1411 features (genes)- # of markers for phenotype **WT**: 849 (60.2% ) with correlation area 60.5%- # of markers for phenotype **NCAPD3\_MUT**: 562 (39.8% ) with correlation area 39.5%- Detailed rank ordered gene list for all features in the dataset- Heat map and gene list correlation  profile for all features in the dataset

#### Global statistics and plots

- Plot of p-values *vs.* NES- Global ES histogram

#### Other

- Parameters used for this analysis

#### Comments

- Timestamp used as random seed: 1599462004429

---

Report: my\_analysis.Gsea.1599462003817.rpt   by user: huisuan1

xtools.gsea.Gsea [Mon, Sep 7, '20 3 PM 0]

Website: www.gsea-msigdb.org/gsea
Questions & Suggestions: Contact page
